# Remembrance of Things Past: Long-Term B Cell Memory After Infection and Vaccination

**DOI:** 10.3389/fimmu.2019.01787

**Published:** 2019-07-31

**Authors:** Anna-Karin E. Palm, Carole Henry

**Affiliations:** Section of Rheumatology, Department of Medicine, University of Chicago, Chicago, IL, United States

**Keywords:** B cell memory, vaccination, mouse vs. human, influenza virus, infection

## Abstract

The success of vaccines is dependent on the generation and maintenance of immunological memory. The immune system can remember previously encountered pathogens, and memory B and T cells are critical in secondary responses to infection. Studies in mice have helped to understand how different memory B cell populations are generated following antigen exposure and how affinity for the antigen is determinant to B cell fate. Additionally, such studies were fundamental in defining memory B cell niches and how B cells respond following subsequent exposure with the same antigen. On the other hand, human studies are essential to the development of better, newer vaccines but sometimes limited by the difficulty to access primary and secondary lymphoid organs. However, work using human influenza and HIV virus infection and/or immunization in particular has significantly advanced today's understanding of memory B cells. This review will focus on the generation, function, and longevity of B-cell mediated immunological memory (memory B cells and plasma cells) in response to infection and vaccination both in mice and in humans.

## Introduction

One of the hallmarks of our immune system is the ability to “remember” past exposure to pathogens. Such exposure can be from infection or vaccination, and by remembering we are, ideally, fully protected from infection upon future encounter with the same pathogen (1). Although humoral immunological memory is mediated in part by serum antibodies secreted by long-lived plasma cells (LLPCs), these cells are usually not described as memory B cells. Instead, memory B cells are defined as long-lived and quiescent cells that are poised to quickly respond to antigen upon recall (2–5).

Both memory B cells and antibody-secreting cells (ASCs) are the product of antigen activation and, most often, interaction with cognate T helper cells. They can be IgM^+^ or immunoglobulin class-switched, and display germline or affinity-matured antigen receptors (B cell receptors; BCRs) (2, 6–8). Although generation of memory B cells does require ligation of CD40 (9), an early burst of both memory B cells and ASCs can form independently of GCs, as well as in T-cell independent responses (10–16). However, T-cell independent memory responses are beyond the scope of this review and will therefore not be thoroughly discussed here.

The terminal differentiation of B cells into ASCs is governed by a gene-regulatory network and modified by environmental stimuli as reviewed in Nutt et al. (17). ASCs can be divided into short-lived ASCs, including short-lived plasma cells and plasmablasts, and LLPCs. Plasmablasts are considered a transient population and can be either precursors of plasma cells (short- and/or long-lived; mainly in mice) or terminally differentiated effector cells activated during ongoing immune responses (mainly in humans) (18–23). In mice, within 2–4 d after infection, plasmablasts are found in extra-follicular zones and differentiate into plasma cells that secrete large quantities of antibodies. This early humoral response of lower affinity usually lasts a few days (24). In contrast, activation and differentiation of B cells within GCs allow the generation of plasma cells of high affinity that will then migrate to the bone marrow, where they can survive for decades and provide long-term humoral protection (25). Such LLPCs are key to maintaining long-term humoral immunity after infection or vaccination. They persist in the absence of antigen for decades after the original exposure (26). Although they exist in multiple lymphoid organs, the bone marrow is the home of the majority of plasma cells in mice (27, 28).

Most of what we know about the generation of plasma cells and memory B cells comes from mechanistic studies in mice. Because of massive differences between mice and humans in terms of life span and cell populations/phenotypes, the biology of mouse and human B cells differs. It is therefore important to also look toward *in vivo* lessons we have learned from humans.

## Lessons From Mouse Studies

### The Plasma Cell vs. Memory B Cell Fate Decision

**Figure 1:**
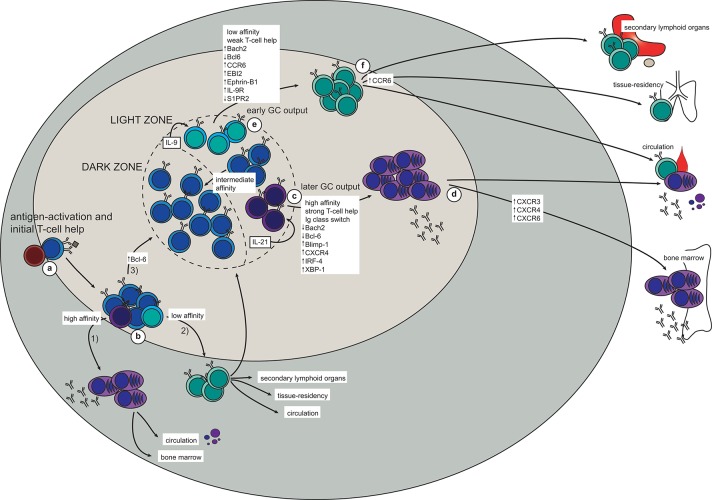
The generation of memory B cells and plasma cells in a T-dependent response (based on mouse studies). **(a)** Antigen-activation brings B- and T cells in contact at the T-B border in secondary lymphoid organs. **(b)** After initial proliferation in the outer follicle, the B cells make their first fate choice: (1) differentiation into extrafollicular (mostly short-lived) plasma cells (higher affinity), (2) differentiation into very early memory B cells (lower affinity), or (3) up-regulation of Bcl-6 and formation of a germinal center (GC). **(c,d)** In the GC, a similar selection process takes place in the light zone (LZ). Here, high-affinity LZ GC B cells receive strong T-cell help and consequently down-regulate Bach2 and Bcl-6 while turning on the plasma cell transcriptional program (Blimp-1, IRF-4, XBP-1; including up-regulation of CXCR4) **(c)**. The plasma cell precursors will then either enter the circulation as short-lived antibody-secreting cells, or they will upregulate CXCR3, CXCR4, and CXCR5 to allow migration to the bone marrow plasma cell niche **(d)**. Here survival factors produced by stromal cells and other adjacent cells (including eosinophils and macrophages) promote their differentiation into long-lived plasma cells, which continue to secrete antibodies for the duration of the lifetime of the host. **(e,f)** Due to the weaker T-cell help received by low-affinity LZ GC B cells, these will not be instructed to turn on either the plasma cell or the GC B cell transcriptional program. Instead, up-regulation of Bach2, CCR6, EBI2, Ephrin-B1, and IL-9R, together with down-regulation of Bcl-6 and S1PR2, promote differentiation to memory B cells **(e)**. To maximize the likelihood of secondary antigen encounter memory B cells will then position themselves strategically in secondary lymphoid organs, become tissue-resident at the site of infection, or patrol as recirculating cells **(f)**.

Following antigen activation with a T-dependent antigen, naïve B cells will interact with cognate T cells at the border between the B- and T-cell zones in the secondary lymphoid organs (Figure 1a). Here, the activated B cells will proliferate and make their first fate decision: whether to differentiate into extrafollicular ASCs or germinal center (GC)-independent memory B cells, or to move deeper into the follicle to form a GC (Figure 1b). A similar choice must then later be made in the light zone (LZ) of the GC, further discussed below. Although the molecular mechanisms for this decision have been extensively studied they have still not been completely elucidated, especially for memory B cell generation. Several studies have addressed the possibility of a “master transcription factor” for memory B cell differentiation, similar to Bcl-6 for GC B cells and IRF-4/Blimp-1 for plasma cells (29, 30). Although Bach2, or specifically high expression of Bach2, in LZ GC B cells has been pointed out as a factor promoting differentiation to memory B cells, a transcription factor unique to memory B cells is yet to be found (31–37). As recently reviewed, ZBTB32, KLF2, ABF-1, and STAT5 have been associated with memory B cell generation, but further studies are needed to understand their role (38).

#### Affinity

There is general consensus in the field that initial affinity for the antigen influences which differentiation pathway will be chosen by an antigen-activated B cell. Newly activated B cells with a relatively high affinity for the antigen will differentiate into short-lived extra-follicular ASCs (39). This ensures that the first burst of secreted antibody has enough affinity for the antigen to opsonize it and form immune complexes that will be directly cleared by phagocytosis, activate complement, and/or be presented on follicular dendritic cells (FDCs), thereby driving affinity maturation in the GC (30, 40). Conversely, antigen-activated B cells of lower affinity typically develop into GC-independent memory B cells. These are most often unmutated and unswitched (IgM+), although class-switched GC-independent memory B cells have been described (13). The GC-independent memory B cells provide a means of retaining adaptability potential within the memory B cell pool, and these cells can either be recruited later in the same response or recalled upon secondary encounter with the antigen.

#### GC Responses

The third fate choice for antigen-activated B cells is to upregulate Bcl-6 and move deeper into the follicle and start a GC reaction [excellently reviewed in Victora and Nussenzweig (30), Mesin et al. (40)]. Briefly, the GC B cells will go through multiple rounds of division in the dark zone (DZ) of the GC, each time introducing mutations in their antigen receptor (B cell receptor; BCR). This process of somatic hypermutation (SHM) leads to affinity maturation and ensures that B cells will specialize their binding to a particular antigen. The mutated B cells will then move to the LZ, where the new BCR will be tested against the antigen presented on FDCs. The B cells that manage to form a BCR with high enough affinity will receive survival signals and either return to the DZ to go through another round of division and SHM, or exit the GC as a plasma cell or a memory B cell.

Similarly to extrafollicular fate decisions, BCR affinity to the antigen seems to play a role also in the GC (37, 41, 42). High-affinity B cells can bind and endocytose more antigen, and consequently present more antigen-derived peptides on class II MHC (MHCII). This higher density of peptide:MHCII on high-affinity B cells gives them an advantage in competing for access to T-follicular helper (Tfh) cells (43–45). In addition, each interaction with a Tfh cell is prolonged and intensified due to a feed-forward loop depending on peptide:MHCII density and CD40:CD40L ligation (45). This enhanced CD40:CD40L interaction causes down-regulation of Bcl6 and turning on of IRF-4 in the GC B cells, allowing them to differentiate into Bcl6^lo^CD69^hi^ plasma cell precursors before exiting the GC as plasma cells (46) (Figure 1c). In addition, IL-21 secreted from Tfh cells is required for plasma cell differentiation (47), further demonstrating the importance of long and strong B:T interactions for this fate decision. A fraction of the plasma cells leaving the GC will home to the bone marrow, where their survival depends on a number of factors in the plasma cell niche (Figure 1d). This will be further discussed below.

Memory B cells, on the other hand, are generated from low-affinity GC B cells in the LZ and will eventually enter the circulation as patrolling cells or take up residence in lymphoid or target organs (Figures 1e,f). The observation that memory B cells consistently are of lower affinity and have fewer mutations than plasma cells indicate that the former are generated before affinity maturation has allowed for the production of high-affinity BCRs. Indeed, an extensive study shows that memory B cells are formed early in the response whereas LLPCs are a later product (15). This temporal discrepancy also fits well with the Bach2 dynamics in memory B cells. Bach2 is required for memory B cell differentiation and only early GC B cells express Bach2, with the expression starting to decline from day 10 (37). Moreover, these experiments show that T cell help, in the form of CD40:CD40L interaction, represses Bach2-expression in GC B cells in a dose-dependent manner. Thus, B cells with higher affinity typically have a lower expression of Bach2 and are therefore predisposed to choose re-entry to the DZ or commitment to the plasma cell transcriptional program. Conversely, relatively weak T cell help, as would be the case for lower-affinity B cells, maintains a relatively high Bach2-expression in LZ B cells, thus favoring a memory B cell fate (37). It is not clear how Bach2 determines memory B cell fate, but it is believed to act as a suppressor of transcription, particularly of *Prdm1* (encoding Blimp-1) and of pro-apoptotic factors such as Bim and Puma (37, 48–51). Thus, it seems likely that lack of strong signaling, and consequently lack of instructions to start the plasma cell or GC B cell transcriptional program forces activated B cells into memory fate. Interestingly, memory B cells and naïve B cells, which are both quiescent with persisting differentiation potential, have similar transcriptional profiles, with the important exception of memory B cells seemingly being hardwired for quick responses (31, 33, 34, 36, 52).

Selection of B cells with a relatively low affinity into the memory compartment early in the response thus ensures that a certain poly-reactivity is maintained within the memory B cell pool. Indeed, preservation of germline, or close to germline, encoded BCRs in memory B cells provides the memory B cell pool with clones that are able to respond quickly while still maintaining a higher degree of flexibility in terms of antigen binding. This flexibility would be lost should only memory B cells with high-affinity mutated BCRs persist in the memory pool. This idea can be illustrated by the observation that around 10% of memory B cells recognize variant antigen better than wild type protein, thus allowing for breadth of protection in a way that LLPCs do not (53). Conversely, by choosing only the highest-affinity GC B cells for plasma cell fate, the quality of the secreted antibodies is ensured to be very high.

#### Immunoglobulin Isotype

Another proposed determinant factor of plasma cell vs. memory B cell differentiation is immunoglobulin isotype. B cells that have switched to IgG, IgE, or IgA are more prone to differentiate to plasma cells than memory B cells (54–58). Interestingly, a recent study showed that even when B cells are forced to switch to IgG1 independently of AID, thus uncoupling the effects of SHM and class-switch recombination (CSR), the switched GC B cells were predominantly differentiating into plasma cells (58). Moreover, transcriptional analysis of IgM^+^ and IgG1^+^ GC B cells in the LZ revealed altered signaling through Nur77 in the switched B cells, associated with increased expression of chemokines associated with exit from the GC into the plasma cell compartment (58). Together, these studies indicate that intrinsic properties of a non-IgM BCR, probably in their signaling capacity, influences the plasma cell vs. memory B cell fate decision.

### Marking Memory B Cell Precursors

Studies aiming at defining memory B cell precursors in the GC have found differential expression of several markers on subsets of GC B cells in the LZ. One such marker is the chemokine receptor CCR6, which has been shown to be dispensable for the initial generation but required for correct positioning of memory B cells as well as for optimal recall responses (59, 60). These CCR6^+^ GC B cells are generally of lower affinity, and have a phenotype closely resembling that of memory B cells (e.g., up-regulated EBI2 and S1PR1, and down-regulated S1PR2) (60). A recent study describes a population of Ephrin-B1^high^S1RP2^low^ GC B cells as memory precursor cells in the LZ, positioned close to the edge of the GC (61). In addition, a study focused on plasma cell precursors in the GC LZ proposes that a fraction of GC B cells in the LZ presenting as Bcl6^low^CD69^low^ are memory B cell precursors (46).

Finally, IL-9R is expressed on memory B cells as well as on a subset of LZ GC B cells concluded to be memory B cell precursors (62, 63). In addition to Bach2-requirement, optimal memory B cell generation also needs Tfh-derived IL-9 (63), and signaling through IL-9R on memory B cells is required for their recall response (64). Taken together, memory B cell precursors may be found in the GC LZ and present as CCR6^+^S1PR2^low^Ephrin-B1^high^Bcl6^low^CD69^low^IL-9R^+^. However, further studies are needed to fully elucidate whether this phenotype really corresponds to a committed memory B cell precursor.

### The Memory B-Cell Niche and Recall Responses

**Figure 2:**
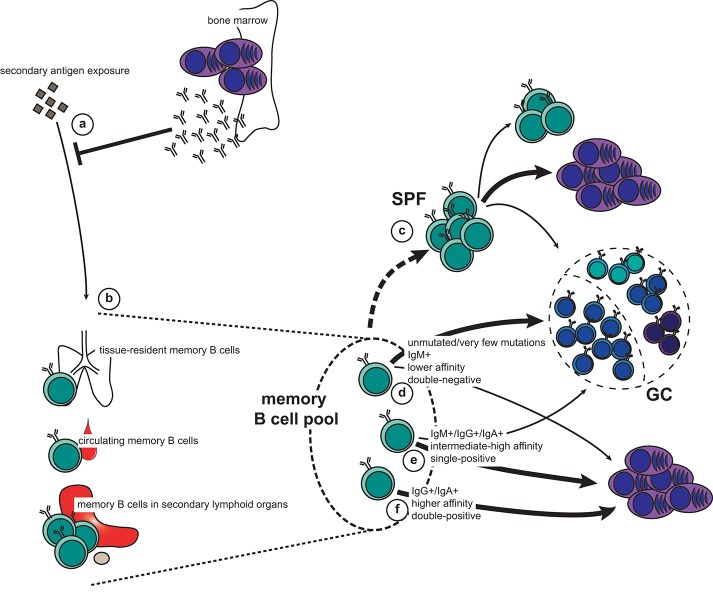
The memory recall response to secondary antigen exposure. **(a,b)** Pre-existing antibodies secreted by long-lived plasma cells (LLPCs) constitute the first line of defense **(a)**. If this is not sufficient for immediate neutralization and elimination of the antigen, memory B cells will be engaged. This can happen either directly in the affected tissue (tissue-resident memory and circulating memory B cells), or when antigen is carried to secondary lymphoid organs **(b)**. **(c)** Activated B cells in lymph nodes can form subcapsular sinus proliferative foci (SPF) upon antigen-dependent re-activation. Although it is unclear which memory subset constitute the SPF, it is known that the main output is plasmablasts, but that this is also the fostering site for new memory B cells as well as cells entering GCs. **(d–f)** Depending on their phenotype, different fate decisions will be made by the reactivated memory B cells: new germinal centers (GCs) are typically formed by IgM^+^, usually unmutated, CD80^−^PD-L2^−^ (double-negative) memory B cells of lower affinity **(d)**. In addition, both IgM^+^ and switched memory B cells that express either CD80 or PD-L2 (single-positive) have retained the capacity to seed GCs **(e)**. However, the bulk of these cells, together with some of the IgM^+^ double-negative memory B cells, will differentiate directly into plasmablasts **(c,d)**. Finally, switched, high-affinity memory B cells that are double positive for CD80 and PD-L2 exclusively form new plasmablasts **(f)**.

Upon re-exposure to an antigen the memory recall response will be faster, stronger, and more specific than a naïve response. Protective memory depends first on circulating antibodies secreted by LLPCs (Figure 2a). When these are not sufficient for immediate pathogen neutralization and elimination, memory B cells are recalled. It is therefore of vital functional importance that memory B cells are stationed at strategic sites where they can maximize their chance of encountering antigen (Figure 2b). The spleen, including the marginal zone, is a major reservoir for memory B cells in both mice and humans (14, 65–67), as is the subcapsular sinus (SCS) of lymph nodes (68). Both the splenic marginal zone and the lymph node SCS are abundant with CD169+ macrophages, which are specialized in presenting unprocessed antigen to B cells (69, 70). It has been demonstrated that both naïve and memory B cells interact with CD169+ macrophages in the SCS, and that upon antigen recall the memory B cells quickly form SCS proliferative foci (Figure 2c), or form new GCs (68). This was also seen in human lymph nodes. Interestingly, the largest output from the SCS proliferative foci is short-lived plasma cells (ASCs), whereas the new GC is a site for further affinity maturation and CSR with very stringent quality controls that limit plasma cell differentiation (42). Importantly, both the SCS proliferative foci and the GC also foster memory B cells that may participate in another re-call response or be recruited later in the same response. In addition to the spleen and lymph nodes, memory B cells are found in the bone marrow, Peyers' patches, gingiva, mucosal epithelium of tonsils, the lamina propria of the gastro-intestinal tract, and in the circulation (67, 71–76). It has not been convincingly demonstrated that the bone marrow, or any other tissue (apart from the spleen and the lymph nodes) contains functional memory B cells or if these memory B cells simply recirculate from the blood to the tissues. These are all anatomical sites where antigen may breach the barriers or be carried to via the circulation, and the memory B cells located here act as sentinels should pre-existing antibodies not provide adequate protection.

Importantly, memory B cells can also seed sites of infection, where they are maintained as tissue-resident memory B cells (77–79). Here they are quickly activated after pathogen invasion without the need for antigen transportation to draining lymph nodes, thus shortening the time for plasma cell differentiation and antibody production on secondary exposure. Interestingly, in the case of influenza virus infection, broadly reactive memory B cells are enriched in the lung-resident pool, thus conferring quick and cross-reactive protection at the site of infection (80).

Upon re-exposure to antigen, memory B cells can quickly proliferate and differentiate into plasma cells. Alternatively, they will re-enter GCs for another round of affinity maturation and CSR. This decision depends on BCR affinity and isotype in addition to differential expression of CD80 and PD-L2 (Figures 2d–f). These surface markers denote functionally different memory B cells independent of immunoglobulin isotype (2, 4, 7, 8, 65). Importantly, the heterogeneity of the memory B cell compartment allows for a functional breadth of memory recall responses.

Unswitched (i.e., IgM^+^) memory B cells are often derived from GC-independent or very early GC responses. They frequently do not express CD80 and/or PD-L2, and carry few, if any, mutations (7, 65). The IgM^+^ memory B cell pool thus keeps a breadth of reactivity similar to that of naïve B cells but with the advantage of being able to rapidly respond to antigen (31, 33, 34, 36, 52). This breadth is particularly important for mounting rapid recall responses to variant antigens, such as influenza virus. On the other hand, recalled IgG+ memory B cells tend to rapidly differentiate into plasma cells without re-entering a GC (4). This is comparable to the fate chosen by switched B cells in the primary GC response (54–58). However, these observations may not be exclusively dependent on immunoglobulin isotype. Indeed, when further dissecting the memory B cell compartment, it becomes apparent that CD80^−^PD-L2^−^ IgM^+^ memory B cells preferentially enter GCs upon recall, whereas those expressing CD80 and/or PD-L2 typically generate rapid IgM^+^ and IgG^+^ plasma cell responses (4, 7, 8, 74, 81). Similarly, IgG^+^ memory B cells single-positive for CD80 or PD-L2 can differentiate to ASCs while retaining the capacity to seed GCs, whereas double-positive IgG^+^ memory B cells only generate ASCs (8). These findings are further supported by studies demonstrating that IgG^+^ and IgA^+^ memory cells can engage in new GC reactions (5, 75).

## Lessons From Human Studies

### Human Plasmablasts

In humans, most studies consider plasmablasts as blood short-lived ASCs generated in acute B cell responses to infection or vaccination that transiently contribute to the serum antibody. In a secondary systemic immune response to a protein antigen such as tetanus toxoid or an inactivated influenza virus vaccine, antigen-specific IgG-secreting plasmablasts with somatically mutated VH gene rearrangements are generated from memory B cells (20, 82). It is also the case following influenza, Ebola, or Dengue virus infection (22, 83–85). It remains an open debate whether human plasmablasts are precursors of and how many do become LLPCs. Evidence suggests that once the infection is cleared, the majority of ASCs undergo apoptosis, while a small proportion may go on to further differentiate into LLPCs (86). The heterogeneity seen in human ASCs from tonsil, blood, and bone marrow reveals stages of increasing maturity, and local profiles of adhesion molecule expression suggest a multi-step model for plasma cell differentiation (82, 87). In human blood when plasmablasts appear between days 6 and 8 after vaccination, they are migratory and attracted by CXCL12 and could migrate to tissues, such as the bone marrow (88, 89).

Plasmablasts have also been described as a “steady state” population where the majority express IgA. They express CCR10 and the adhesion molecule β_7_ integrin and they are attracted by CXCL12 suggesting that they come from mucosal immune reactions and can return to mucosal tissue. Approximately 40% of LLPCs in human bone marrow are IgA^+^, non-migratory, and express β_7_ integrin and CCR10, suggesting a substantial contribution of mucosal plasma cells to bone marrow resident LLPCs (90). After tetanus vaccination, IgG^+^${\text{CD62L}}^{+}\beta_{7}$ integrin^−^ dividing, vaccine-specific, and migratory plasmablasts appear in the blood, as do non-dividing, non-migratory, CD62L^−^ plasma cells of different specificities (90).

A recent study identified survival factors from the bone marrow niche that favors maturation of human blood ASCs to LLPCs *in vitro* (91). IL-6 and two members of the tumor necrosis factor (TNF) superfamily: BAFF (B-cell activating factor of the TNF family; also known as BLyS in humans) and APRIL (a proliferation-inducing ligand) are known to be important survival signals (92), as well as is CXCL12 (93). Additional factors secreted by the bone marrow niche such as fibronectin and YWHAZ are important for LLPC maturation (91).

### LLPCs

#### Migration to and From the Bone Marrow

Human LLPCs freshly isolated from the bone marrow have high expression of the chemokine receptors CXCR4 and CXCR6 and responsiveness in *in vitro* migration assays to the chemokines CXCL12 and CXCL16. The chemokine CCL28 has also been shown to attract human bone marrow plasma cells *in vitro* (94). Two interesting populations have been observed in the blood of tetanus toxoid immunized individuals: a population of migratory plasmablasts expressing CXCR3 and CXCR4, and a population resembling mature plasma cells of the bone marrow. These findings suggest that these cells are likely to be resident LLPCs mobilized from their survival niches in the bone marrow, in competition with newly generated plasmablasts (88).

#### In the Bone Marrow

Mesenchymal stromal cells (MSC) in the human bone marrow microenvironment provide factors that support LLPC survival (95–97). Cytokines of the TNF superfamily (BAFF, APRIL and TNF-α), IL-6 family, CD80/CD86, CD44 binding to hyaluronic acid, and VLA-4 binding to VCAM-1/fibronectin promote survival of plasma cells. CXCL12 promotes entry of cells to the bone marrow as well as plasma cell survival (86). BAFF seems to be important for human plasmablast differentiation whereas APRIL is the key to long-term survival in the bone marrow (98). An interesting study demonstrated that extracellular vesicles from bone marrow-derived MSCs support *ex vivo* survival of human ASCs (99).

In humans, the bone marrow contains both CD19^+^ and CD19^**−**^ LLPCs (26). The majority of CD19^**−**^ LLPCs are actually found in the bone marrow, compared to the blood, spleen and tonsils. Interestingly, CD19^**−**^ LLPCs are enriched in IgG^+^ cells and carry fewer VH mutations compared to CD19^+^ LLPCs. Only CD19^**−**^ LLPCs resist to mobilization into the blood following immunization, and are resistant to depletion by Rituximab. In addition, CD19^**−**^ LLPCs were not found in the bone marrow of 5–7 months old infants while CD19^+^ LLPCs were present. This study suggests a multi-layer model of LLPCs in the human bone marrow with CD19^+^ LLPCs being a dynamic component and CD19^**−**^ a more static component permitting both adaptation and stability of humoral protection (100). A more recent study of the same populations but this time in response to influenza virus vaccination suggests that newly generated ASCs can acquire a mature plasma cell phenotype that is accompanied by loss of CD19 expression at an early stage of differentiation, and that aging is not an obligate requirement for a CD19^**−**^ state to be established (101). Finally both CD19^+^ and CD19^−^ vaccinia-specific LLPCs were detected in the BM more than 35 years after the eradication of smallpox, suggesting that the LLPC pool may be maintained by a process in which vaccinia-specific B cells differentiate into LLPCs in the BM (26).

#### Outside of the Bone Marrow

Compared to the bone marrow niche, fibroblasts from the lymph nodes and the spleen have been poorly characterized in both mice and humans. A few studies have shown that stromal cells in the spleen and lymph nodes might promote plasma cell survival *in vitro* (102, 103). Recently, a new subset of fibroblasts (FRCs for fibroblastic reticular cells) in the lymph nodes have been described both in mice and humans as the main cell type in contact with plasma cells to guide them in their migration (104).

### Mucosal Plasma Cells

Plasma cells are very abundant in mucosal tissues. They are located both in the connective tissue (lamina propria) and in lymphoid organs such as the tonsils in the oral cavity and Peyer's patches in the gut. The majority of these plasma cells secrete IgA antibodies, and humans also have a substantial IgM^+^ plasma cell population in the mucosa (105). B cells in the respiratory tract and IgA responses in the gastrointestinal tract in have been nicely reviewed in Kato et al. (106) and Bunker and Bendelac (107), respectively, and are both beyond the scope of this review.

### Human Memory B Cells

A great variety of B cell subsets have been identified in the tonsil, spleen, and peripheral blood and represent different stages of development of a naive B cell into a memory B cell. In the human tonsil, at least five distinct subpopulations of mature human B cells (Bm1–Bm5) have been identified. Concisely, naive B cells belong to the Bm1 and Bm2 subpopulations whereas fully differentiated memory B cells belong to the Bm5 subset (108–110). Interestingly IgG transcripts in the tonsil had accumulated twice as many mutations as the IgM transcripts suggesting that reentry of selected B cells in the GC to generate higher affinity BCRs is a possibility (109).

As we previously stated, memory B cells are mainly generated in the GCs in secondary lymphoid organs. After leaving the GCs, memory B cells either join the recirculating pool of lymphocytes, or home to antigen draining sites. Memory B cell niches outside of the blood have been described and memory B cells have been found in the bone marrow, the tonsil and the spleen (111). Additionally a population of tissue based memory B cells expressing Fc receptor-like 4 (FCRL4) instead of CD27 has been described (112, 113). In the blood and bone marrow, human memory B cells can be divided in three main populations: CD19^+^CD27^+^IgM^+^IgD^+^ (similar to marginal zone (MZ) B cells), CD19^+^CD27^+^IgM^+^IgD^−^ (IgM-ONLY) and class-switched CD19^+^CD27^+^IgM^−^ (IgG^+^ or IgA^+^) (114, 115). An in-depth flow cytometry analysis of human bone marrow and blood samples showed that compared to the blood, the bone marrow was enriched in both MZ and switched B-cells (116). In the spleen, two main phenotypically distinct B cell populations exist and localize to separate areas of the lymphoid tissue. Mantle zone B cells (IgD^high^IgM^+^CD21^+^CD23^+^) are unmutated and believed to be naive B cells, whereas MZ B cells are IgD^+^IgM^high^CD21^high^CD23^±^ and exhibit somatic mutations (117–119). It has been demonstrated that CD148, as well as CD27, are markers for memory B cells present in the human spleen (120). More recently, a population of IgG^+^ memory B cells residing in the MZ of the spleen have been found and examined. IL-21 and BAFF have been demonstrated to be important for the differentiation of these IgG^+^ splenic memory B cells into plasma cells (121).

#### CD19^+^CD27^+^IgM^+^IgD^+^ (Also Called Human MZ B Cells)

The spleen is an important organ in the defense against encapsulated bacteria. A population of “IgM memory B cells” controlling *Streptococcus pneumoniae* is observed in the spleen (122). Additionally, the human peripheral B-cell compartment displays a large CD19^+^CD27^+^IgM^+^IgD^+^ memory B cell population, resembling the splenic MZ B cells. In fact, by CDR3 spectratyping and gene-expression profiling, it has been demonstrated that CD19^+^CD27^+^IgM^+^IgD^+^ memory B cells are circulating splenic MZ B cells. These memory B cells have a mutated BCR, provide a pre-diversified immune repertoire and are involved in T-independent responses (123). They can develop in the absence of a spleen, but splenectomy in older individuals dramatically reduces the number of blood MZ B cells (122, 124). Finally, when compared to switched memory B cells in children <2 year of age, CD27^+^IgM^+^IgD^+^ memory B cells in the spleen and blood do not display any signs of antigen-driven activation and expansion despite the many antigenic challenges experienced during childhood, suggesting a developmental diversification outside of T-dependent and T-independent responses (125).

#### CD19^+^CD27^+^IgM^+^IgD^−^ (IgM-Only) and Class-Switched CD19^+^CD27^+^IgM

By tracking tetanus toxoid-specific memory B cells (CD3^**−**^CD19^+^CD20^+^CD27^+^) at steady state, it has been showed that the spleen is the largest reservoir of memory B cells followed by the tonsil. Bone marrow and blood memory B cells express surface IgG and IgA at similar frequencies, while the tonsil contained more IgA memory B cells compared to other locations. IgG^+^ memory B cells were enriched in the spleen and the tonsil compared to the bone marrow and the blood and IgM^+^IgD^+^ memory B cells were reduced in the tonsil compared to other locations. Interestingly, the absence of spleen and tonsils does not affect secondary responses to tetanus, suggesting an organ independent maintenance and reactivation for human memory B cells (111). Memory B cells that reside in lymphoid organs and recirculate after re-exposure to antigen are phenotypically the same and do not represent different stages of maturity. Additionally, it has been demonstrated that the human spleen is a major reservoir of long-lived vaccinia-specific memory B cells (66). Indeed, anti-smallpox IgG^+^ memory B cells were specifically enriched in the spleen, confirming that the spleen is a major reservoir for long-lived memory B cells.

Finally, high-throughput VH sequencing on paired blood and spleen samples revealed that IgM sequences from clones shared between the MZ and the memory IgG/IgA (switched) compartments displayed a mutation and repertoire profile of IgM-only and not of MZ B cells. Thus the “IgM-only” subset appears as the only subset showing precursor–product relationships with CD27^+^ switched memory B cells, indicating that they represent GC-derived IgM memory B cells and that IgM-only and MZ B cells constitute two distinct entities (126).

### Human IgG and IgA Responses Induced by Infection and Vaccination

The route by which an antigen enters the body (systemic vs. mucosal) and the nature of the antigen are factors that direct the immune response class-switching patterns. Protein antigens usually trigger B cells receiving T-cell help while polysaccharide antigens induce CSR in the absence of T-cell help. Moreover, BAFF and APRIL have been shown to stimulate CSR to IgG and IgA in human B cells (127). Polysaccharide B cell responses to vaccination in humans have been reviewed in Mitchell et al. (23), while the kinetics of ASC responses to infection have been reviewed in Carter et al. (128).

#### IgG

Antibody responses to soluble protein antigens and membrane proteins primarily induce IgG1, but are accompanied with lower levels of the other subclasses. Viral infections in general lead to IgG antibodies of the IgG1 and IgG3 subclasses (129). On the other hand, antibody responses to bacterial capsular polysaccharide antigens is almost only restricted to IgG2 (130). IgG4 antibodies are often formed following repeated or long-term exposure to antigen in a non-infectious setting (131).

#### IgA

Homeostatic IgA responses employ a polyreactive repertoire to bind to a broad subset of microbiota species and tend to be of low affinity. In contrast, mucosal pathogens and vaccines elicit high-affinity, T-cell dependent antibody responses (107, 132). Mucosal IgA responses through a T-cell dependent reaction that place in mucosal lymphoid follicles, such as intestinal Peyers' patches and mesenteric lymph nodes (together called MALT for Mucosa-Associated Lymphoid Tissues) (132). Human IgA subtypes show distinct anatomical expression patterns, with monomeric IgA1 dominating in the serum and dimeric IgA2 in the gut (133).

Very few studies in humans have compared the induction of IgA and IgG secreting cells following various routes of immunization. An early study compared oral, intranasal and systemic influenza virus vaccines in healthy adults. Both systemic and intranasal immunizations induced mainly IgG^+^ influenza-specific B cells in the blood after vaccination while the oral route induced IgA^+^ influenza-specific B cells in the blood. Additionally, oral and intranasal administration of antigen-induced IgA influenza-specific antibodies in external secretions (134). These results were confirmed later on by multiple studies reporting a bursting population of IgG^+^ antigen-reactive plasmablasts in the blood after secondary tetanus toxoid vaccination (88), influenza virus vaccination or infection (20, 83, 135), as well as acute dengue virus infection (22). In addition, immunization of African green monkeys with a live-attenuated H5N1 influenza vaccine resulted in more serum IgG neutralizing antibodies than IgA (136).

A study employing Ad26/Env (HIV) vaccination in rhesus macaques demonstrated highly coordinated IgG and IgA responses in both peripheral blood and mucosal compartments (137). It remains unclear to this day how related IgG and IgA plasmablasts/plasma cells are and what the relationship between mucosal and systemic antibody responses looks like. While a study suggested that mucosal and systemic humoral immune responses are regulated independently of each other based on the observation that systemic vaccination does not seem to impact peripheral IgA^+^ plasmablast numbers (90, 138), another study revealed that in celiac disease patients, the same antigen-reactive B cell clones that give rise to gut plasma cells also contribute to the serum IgG and IgA antibody pool. However, serum IgA antibodies had a molecular composition (IgA1 vs. IgA2 and J chain level) distinct from that of IgA antibodies secreted in the gut, suggesting the involvement of different plasma cell populations (139). Finally, analysis of long-term transcriptional profile between blood IgG and IgA influenza-reactive plasmablasts as well as influenza-negative IgA plasmablasts did not reveal any specialization based on isotype. These data suggest that IgG and IgA vaccine–positive plasmablasts are largely similar, whereas IgA vaccine–negative plasmablasts appear to be transcriptionally distinct from antigen-induced peripheral blood plasmablasts (140).

### Lessons From HIV

Significant efforts in the HIV field are focusing on the design of vaccines that would induce the generation of broadly neutralizing antibodies (bNAbs). Understanding the immunology behind the development of antibody potency and breadth following immunization is crucial in this context, not only to the HIV community (141). The success of most vaccines relies on the generation of antibodies to provide protection against subsequent infection. As discussed earlier in this review, Tfh cells are critical for the production of high-affinity B cell clones in the GC and thus the generation of long term memory, i.e., memory B cells and LLPCs (142).

The feasibility of assessing GCs and Tfh responses from human lymph nodes has been limited, as GC B cells do not circulate in the blood, and lymph nodes are rarely sampled (143). Recently, fine needle aspirates of the draining lymph nodes were used to longitudinally sample GC B cells and GC Tfh cells in non-human primates. The lymph node fine needle aspiration technique has proven effective in terms of how many cells were recovered from the biopsy as well as in not disrupting the ongoing GC. The authors found that neutralizing antibodies in non-human primates correlate with GC B cell magnitude and Tfh help quality (144). They also found that GCs peak weeks after the initial immunization. This means that a classic immunization (one injection of antigen) is not optimal for “feeding” the peak GC response. Proteins that are not of extreme stability can be degraded, exposing epitopes that would normally be hidden or non-existent on a more native protein conformation. Slow immunogen release could improve the availability of intact antigen and epitopes of interest for the duration of the GC response (145).

Germline-targeting strategies aim to activate B cell precursors with potential interest for bNAbs generation, so that they will enter the GC, be selected and affinity matured and will generate memory B cells. Studying HIV-reactive B cell lineages to infer unmutated ancestral BCRs that represent what a naïve B cell would express is the key to a B-cell lineage vaccine strategy (146). A vaccination protocol based on B-cell lineage differs from classic protocols in the fact that they may prime with one immunogen and boost with another or with a sequence of several different immunogens (147–150).

It has been recently demonstrated that only immunogens above a certain affinity and in multimeric form are capable of inducing GCs dominated by B cells from a bNAb precursor starting with low precursor frequency (151). These B cells successfully competed in GCs, underwent somatic hypermutation and differentiated into memory B cells. Overall this study demonstrates that germline-targeting immunogens can overcome affinity, avidity, and inter-clonal GC competition challenges with high-affinity multimeric designs.

### Lessons From Influenza

Plasmablasts have been extensively studied in humans, especially in the context of influenza vaccination and infection. Little is known about B cells that become activated but do not differentiate into plasmablasts. A subset of antigen-reactive B cells called ABCs for “Activated B Cells” has been described and was found to be transcriptionally distinct from the ASC population and committed to the memory lineage (152). ABCs and ASCs share hemagglutinin (HA)-reactive clones following influenza vaccination. Our laboratory also described a post-GC population of B cells that phenotypically resemble memory B cells but that have low expression of CD21 (classical memory B cells are CD19^+^CD27^+^CD21^high^). We demonstrated that the CD21^low^ population was comprised of recent GC graduates that were refractory to GC reentry and seemed to be predisposed to differentiation into long-lived plasma cells (153). Although clonally related to memory B cells and plasmablasts, CD21^low^ B cells form distinct clades within phylogenetic trees based on the accumulation of variable gene mutations. Another study demonstrated that HA-reactive CD21^low^ B cells are enriched in the blood compared to the tissues while there was an enrichment of CD27^+^CD21^high^HA^+^ B cells in all tissues. Both CD21^+^ and CD21^low^ populations were not maintained in the peripheral blood at 1 year post-vaccination (154).

Additionally, it is of great interest to understand how different vaccine compositions will affect the generation of memory B cells and LLPCs. Seasonal influenza vaccines exist as live-attenuated influenza virus (LAIV), which more closely resembles natural immunity after infection, or as inactivated vaccines. LAIV have been used mostly in children but do not induce strong systemic antibody responses in adults (155). The same was true for two different avian pandemic LAIV vaccines (H5N1, H7N9), although these vaccines elicited a long-term immune memory that was revealed after administration of a matched inactivated vaccine (156–158). To understand how LAIV vaccines can prime such a memory response, a detailed analysis of B cell responses in systemic and local lymphoid tissues in a non-human primate model was performed (136). Interestingly, the authors found that the LAIV vaccine induced robust GCs in the mediastinal (lung-draining) lymph node and that both HA-reactive plasmablasts and memory B cells were found in the mediastinal lymph nodes after immunization.

Finally, it is believed that adjuvants can modulate humoral responses and retain antigen at the site of injection. Most studies have been done with alum and it remains unknown how other adjuvants (such as AS03 and MF59) act on GCs and antigen release (159). In the context of influenza vaccines, adjuvanted vaccines administered in patients with impaired immune responses, such as infants and the elderly, were shown to be beneficial (160–162). Additionally a study showed that the adjuvant AS03 induced an increased activation of naïve B cells and an increased adaptability of recalled memory B cells, improving immunogenicity (163).

## Conclusion

The generation of memory B cells and long-lived plasma cells is crucial to the long-term effectiveness of vaccines. Understanding how to induce these different populations and modulate their effects both in animal models and human is essential to the design of better vaccines. Thus, the design of new immunogens, how to release them, as well as the mechanisms of actions of various adjuvants are the future of vaccines protecting against challenging or emerging infectious diseases.

## Author Contributions

A-KP and CH contributed ideas and wrote the review. A-KP designed the figures.

### Conflict of Interest Statement

The authors declare that the research was conducted in the absence of any commercial or financial relationships that could be construed as a potential conflict of interest.
